# Spatial Non-Cyclic Geometric Phase in Neutron Interferometry

**DOI:** 10.6028/jres.110.034

**Published:** 2005-06-01

**Authors:** Stefan Filipp, Yuji Hasegawa, Rudolf Loidl, Helmut Rauch

**Affiliations:** Atominstitut der Österreichischen Universitäten, Stadionallee 2, A-1020 Vienna, Austria; Institut Laue Langevin, Boîte Postale 156, F-38042 Grenoble Cedex 9, France; Atominstitut der Osterreichischen Universitaten, Stadionallee 2, A-1020 Vienna, Austria; Atominstitut der Osterreichischen Universitäten, Stadionallee 2, A-1020 Vienna, Austria; Institut Laue Langevin, Boîte Postale 156, F-38042 Grenoble Cedex 9, France; Atominstitut der Österreichischen Universitäten, Stadionallee 2, A-1020 Vienna, Austria

**Keywords:** geometric phase, neutron interferometry

## Abstract

We present a split-beam neutron interferometric experiment to test the non-cyclic geometric phase tied to the spatial evolution of the system: the subjacent two-dimensional Hilbert space is spanned by the two possible paths in the interferometer and the evolution of the state is controlled by phase shifters and absorbers. A related experiment was reported previously by some of the authors to verify the *cyclic* spatial geometric phase. The interpretation of this experiment, namely to ascribe a geometric phase to this particular state evolution, has met severe criticism. The extension to *non-cyclic* evolution manifests the correctness of the interpretation of the previous experiment by means of an explicit calculation of the non-cyclic geometric phase in terms of paths on the Bloch-sphere. The theoretical treatment comprises the cyclic geometric phase as a special case, which is confirmed by experiment.

## 1. Introduction

Since the discovery of a geometric effect by Berry [1] in the shape of an additional phase factor after an adiabatic and cyclic transport of a quantum system, Berry’s phase has been intensively investigated and generalized: the extension to degenerate subspaces by Wilckzek [2], the removal of the adiabatic constraint by Aharonov and Anandan [3] and the cyclic condition by Samuel and Bhandari [4] using the early ideas of Pancharatnam [5] and the kinematic approach to geometric phases by Mukunda and Simon [6], to name a few. In all theses contexts the geometric phase is dependent only on the geometry of the subjacent Hilbert space, but not on the particular dynamics of the system under consideration. Furthermore, Manini and Pistolesi [7] proposed an off-diagonal geometric phase to exhibit the geometry of state space in situations where the usual (diagonal) geometric phase is undefined. This has been verified experimentally by some of the authors [8].

In the course of the development of quantum mechanics it has become clear that the concept of pure states is not sufficient when taking environmental influences causing decoherence effects into account. Then one has to use the concept of mixed states. Probably the first treatise of a geometric phase for mixed states is due to Uhlmann [9] in a quantum algebraic context. Another definition of a mixed state geometric phase is due to Sjöqvist et al. [10] using an interferometric approach for its definition. For these concepts one has to keep in mind that there exist points in parameter space for which the mixed state geometric phases remain undefined provoking an extension to off-diagonal mixed state geometric phases [11].

The geometric phase is associated with an evolution of a system governed by an Hamiltonian, e. g., a neutron in a magnetic field where the geometric phase arises by the spinor evolution due to the coupling with the magnetic field. Here, we observe a geometric phase as an effect of the change in the spatial degrees of freedom in an interferometry setup. A proposal to verify the spatial geometric phase is due to Sjöqvist [12] using polarized neutrons by reversing the roles of the magnetic field and the spatial degrees of freedom. Moreover, an experiment using unpolarized neutrons has been performed by Hasegawa et al. [13, 14] to test the cyclic spatial geometric phase by inducing a relative phase shift of 2π between the interfering neutron beams in a perfect silicon single-crystal interferometer. The geometric interpretation of this experiment has been dismissed by Wagh [15] demanding further investigations, namely in the non-cyclic case, which is the purpose of the current article.

## 2. Geometric Phases

Let us briefly review the basic concepts of geometric phases: A geometric phase is a quantity which is deeply connected to the curvature of some underlying (state- or parameter-) space. A two-dimensional plane in three-dimensional real space does not have an intrinsic curvature, but when considering a sphere embedded in euclidean real space, we have to take the curvature of this manifold into account. In geometry this curvature is reflected, for example, in the angle difference of a vector transported around a loop along geodesics, i. e., great circles: If a vector is pinned onto a sphere and then transported along a meridian to the equator, for some angle α along the equator and back to the initial point without changing its length and its direction in the tangent plane to the surface of the sphere, the vector will point in a different direction with a relative angle of α as the holonomy associated with the loop. If we do the same on a two-dimensional plane the initial and the final vector will point in the same direction.

Berry [1] was the first who addressed this issue in quantum mechanics: He considered a system initially in an eigenstate |*n*(***R***(*t*))〉*_t_*_= 0_ of the governing Hamiltonian *H*(***R***(*t*)) dependent on the parameters ***R***(*t*) changing with time *t*. As a demonstrative example one may consider a neutron coupling to a magnetic field *H*(***R***(*t*)) = −***µ*** · ***B***(***R***(*t*)) due to its magnetic dipole moment ***µ*** = *µ*_n_***σ***, where ***σ*** = {*σ_x_*, *σ_y_*, *σ_z_*} are the Pauli matrices and *µ*_n_ denotes the magnetic moment of a neutron. Suppose now that the neutron is initially polarized in the direction of the magnetic field. If the direction of the magnetic field is changed adiabatically, i. e., slowly enough to avoid transitions to an orthogonal state, the system will stay in the eigenstate |*n*(***R***(*t*))〉 at all times *t*. Furthermore, when tracing out a *loop* in parameter space the final state |*Ψ* (*τ*)〉 at time *τ* will be the same as the initial state up to an additional phase factor:

$$\begin{array}{c} |\Psi \left(\tau \right)\rangle =e^{i\phi d}e^{i\phi g}|n\left(R(\tau )\right)\rangle \\ \;=e^{-\frac{i}{h}\int_{0}^{\tau }E_{n}(t)dt}e^{-\oint_{C}\langle n(R)|\nabla_{R}|n(R)\rangle dR}|n\left(R(\tau )\right)\rangle . \end{array}$$ (1)

The first phase value $\phi_{d}=\frac{1}{\hbar }\int_{0}^{\tau }E_{n}\left(R(t)\right)dt$ is dependent on the time needed to traverse the loop and on the instantaneous energy *E*_n_(*t*) = 〈*Ψ*(*t*)|*H*(*t*)|*Ψ*(*t*)〉 of the system, whereas the second phase *ϕ*_g_ = *i*·∮_C_ 〈*n*(***R***)|∇*_R_*|*n*(***R***)〉d***R*** is dependent only on the circuit integral in parameter space revealing the geometric structure. The latter is termed *Berry phase* or more general *geometric phase* in contrast to the former *dynamical phase ϕ*_d_. *ϕ*_g_ can be rewritten as a surface integral by use of Stoke’s Theorem yielding *ϕ_g_* = −Im ∫_F_ d***SV***_n_(***R***), where *F* is the surface enclosed by the loop in parameter space with d***S*** denoting the area element and *V*_n_ = *∇* × 〈*n*|*∇n*〉 in an obvious abbreviated notation. For the neutron example—or more generally for any spin-1/2 particle—*ϕ*_g_ equals half of the solid angle enclosed by the loop as seen from the degeneracy point |***R***| = 0 in parameter space. This can also be related to the example from geometry above where the holonomy after the transport of the vector pinned initially to the north pole of a sphere equals the solid angle as seen from the origin of the sphere.

Several restrictions have been relaxed in course of the years, e. g., extensions to nonadiabatic [3], noncyclic and nonunitary [4], and nonpure [9, 10] geometric phases have been made. Important in our case are the generalizations to the nonadiabatic regime by Aharonov and Anandan and to noncyclic paths by Samuel and Bhandari.

In this case we have to introduce the Projective Hilbert space (Ray space) ℛ by identifying all state vectors in Hilbert space ℋ which differ only by an overall phase factor:

$$|\phi \rangle \sim |\phi^{\prime }\rangle :|\phi^{\prime }\rangle =e^{i\alpha }|\phi \rangle ,\alpha \in \mathbb{R}.$$ (2)

The stress is therefore shifted from the parameter space of the Hamiltonian in case of Berry’s construction to state space. We are not interested in the changes of the driving parameters (as the direction and strength of the magnetic field) but in the changes of the state itself. In Berry’s considerations these two spaces are identical since the state follows the changes in parameters due to the adiabaticity condition.

In the construction by Aharonov and Anandan one considers an open path in Hilbert space which is projected to a path in Ray space by use of the equivalence relation in Eq. (2), i. e., the curve C:t∈[0,τ]→|ϕ(t)〉∈ℋ is projected to $\tilde{C}:t\in [0,\tau ]\to \pi (|\phi (t)\rangle )\equiv |\phi (t)\rangle \langle \phi (t)|\in ℛ$ with |*ϕ* (0)〉 ~ |*ϕ* (*τ*)〉. For $\tilde{C}$ an absolute phase factor of |*ϕ* (*t*)〉 is immaterial, since the curve $\tilde{C}$ is defined via the evolution of the projection operator |*ϕ* (*t*)〉 〈*ϕ* (*t*)|. The geometric phase is a property of Ray space, where $\tilde{C}$ is closed due to the equivalence of the initial and the final state |*ϕ* (0)〉 ∼ |*ϕ* (*τ*)〉 and can be calculated via a surface integral over the area enclosed by $\tilde{C}$.

One can find many different curves *C*′, *C*″, … in Hilbert space differing by a phase factor e^iα(t)^ and yielding the same curve in Ray space under the projection map π. On the other hand for a given curve in Ray space, there exists one distinct curve in Hilbert space fulfilling the parallel transport conditions, namely that two neighbouring states |*ϕ* (*t*)〉 and |*ϕ* (*t +* d*t*)〉 in ℋ have the same phase, that is to say, 〈*ϕ* (*t*)|*ϕ* (*t +* d*t*)〉 is real and positive. This implies by Taylor expansion that $\langle \phi (t)|\frac{d}{dt}|\phi (t)\rangle =0$ [18]. For this curve the dynamical phase vanishes as one can verify by inserting the Schrödinger equation *H*(*t*)|*ϕ*(*t*)〉 = *iħ*d/d*t*|*ϕ*(*t*)〉 into the parallel transport condition.

The concept can be extended to apply to open paths in Ray space where |*ϕ* (*τ*)〉 ≁ |*ϕ*(0)〉 by closing the curve by a geodesic, i. e., a path in Ray space with the shortest distance from |*ϕ* (*τ*)〉 〈*ϕ* (*τ*)| to |*ϕ* (0)〉 〈*ϕ* (0)|. Then one obtains a well-defined surface area enclosed by the path generated by the evolution of the system plus the geodesic closure. This surface provides an expression for the geometric phase, which has been proven by Samuel and Bhandari [4].

To sum up, for a general evolution of a quantum state the state obtains a dynamical phase dependent on the energy and time as well as a geometric phase only dependent on the subjacent geometry of state space. For special Hamiltonians which fulfill the parallel transport conditions the dynamical phase vanishes, which is also the case when the state is transported along a geodesic. An example of the latter is an evolution along a great circle on a sphere for a two-level system which we will encounter in the forthcoming discussion.

## 3. Interferometric Setup

Due to Feynman [16] the description of any two-level quantum system is equivalent to the description of a spin-1/2 particle. Exploiting this equivalence there is in principle no difference between manipulations in the spin space of neutrons with the orthogonal basis {|↑〉, |↓〉} as eigenstates of *σ*_z_, and momentum space with {|*k*〉, |*k*′〉} as orthogonal basis vectors corresponding to two directions of the neutron beam in an interferometer. In both cases one can assign a geometric phase to the particular evolution of the initial state. An even more appropriate description for the interferometric case for the forthcoming discussion is in terms of “which-way” basis states {|*p*〉, |*p*^⊥^〉}, namely, if the neutron is found in the upper beam path after a beam-splitting plate it is said to be in the state |*p*〉, or in the state |*p*^⊥^〉, if found in the lower beam path. In case of a 50 : 50 beamsplitting of the incident (neutron) beam into a transmitted beam and a reflected beam, the associated wave vector after the beamsplitter can be written as an equally weighted coherent superposition of the two paths $|q(\delta )\rangle \equiv 1/\sqrt{2}\left(|p\rangle +e^{i\delta }|p^{⊥}\rangle \right)$ with the relative phase δ ∈ ℝ depending on the particular physical reaization of the beamsplitter.

**Fig. 1:**
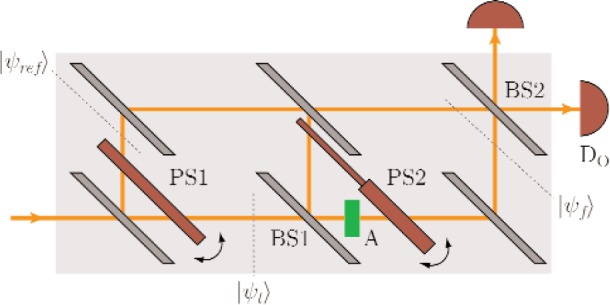
Experimental setup to test the spatial geometric phase in a neutron interferometer.

For testing the spatial geometric phase we use a double-loop interferometer (Fig. 1), where the incident unpolarized neutron beam |*ψ*〉 is split up into a diffracted reference beam |*ψ*_ref_〉 and a transmitted beam |*ψ*_t_〉. The transmitted beam is subjected to further evolution in the second loop of the interferometer by use of beam-splitters (BS1 and BS2), an absorber (A) with transmission coeficient *T* and the phase shifter PS2 generating a phase shift of e*^iϕ^*^1^ on the upper and e*^iϕ^*^2^ on the lower beam path, respectively, yielding the final state |*ψ*_f_〉 = *U* |*ψ*_t_〉 = *U* |*p*〉. Here |*ψ*_t_〉 = |*p*〉 since before the beam splitter BS1 the beam is clearly localized as seen from the second loop of the interferometer. The unitary matrix *U* = *U*(*T*, *ϕ*_1_, *ϕ*_2_) comprises all the manipulations in the second loop:

$$\begin{array}{r} |\psi_{t}\rangle \overset{\text{BS}}{\to }\frac{1}{\sqrt{2}}(|p\rangle +|p^{⊥}\rangle )\overset{A}{\to }\frac{1}{\sqrt{2}}(|p\rangle +\sqrt{T}|p^{⊥}\rangle )\\ \overset{\text{PS}2}{\to }\frac{1}{\sqrt{2}}(e^{i\phi 1}|p\rangle +\sqrt{T}e^{i\phi 2}|p^{⊥}\rangle )\equiv U|\psi_{t}\rangle =|\psi_{f}\rangle . \end{array}$$ (3)

The geometric phase can then be extracted from the argument of the complex valued scalar product between the initial and the final state arg 〈*ψ*_t_|*ψ*_f_〉 (when removing dynamical contributions as will be discussed later). This is where the reference beam comes into play: |*ψ*_ref_〉 is not subjected to any further evolution, but is stationary apart from adding a phase factor e^iη^ by use of the phaseshifter PS1. |*ψ*_ref_〉 propagates towards the beamsplitter BS2 from the upper path, thus we can assert it to be in the state e^iη^|*p*〉. Then by the variable phase shift e^iη^ one can measure the shift of the interference fringes reflecting the phase difference between |*ψ*_ref_〉 and |*ψ*_f_〉.

This preparation of the states is followed by the recombination of the two beams |*ψ*_f_〉 and |*ψ*_ref_〉 at the beamsplitter BS2 and the detection at the detector *D*_0_ in the forward beam. This step can be described by the application of the projection operator |*q*〉 〈*q*| = 1/2(|*p*〉 + |*p*^⊥^〉) (〈*p*| + 〈*p*′|) (with *δ* = 0, which can always be achieved by an appropriate choice of the phase of the basis states) to |*ψ*_f_〉 as well as to |*ψ*_ref_〉:

$$\begin{array}{l} |{\psi^{\prime }}_{f}\rangle =|q\rangle \langle q|\psi_{f}\rangle =K(e^{i\phi 1}+\sqrt{T}e^{i\phi 2})|q\rangle \\ |{\psi^{\prime }}_{\text{ref}}\rangle =|q\rangle \langle q|\psi_{\text{ref}}\rangle =K|q\rangle , \end{array}$$ (4)

where *K* is some scaling constant.

The intensity *I* measured in the detector *D*_0_ is proportional to the absolute square of the superposition $|{\psi^{\prime }}_{f}\rangle +e^{i\eta }|{\psi^{\prime }}_{\text{ref}}\rangle$:

$$\begin{array}{l} I\propto {\left|(e^{i\eta }+e^{i\phi 1}+\sqrt{T}e^{i\phi 2})|q\rangle \right|}^{2}=\langle {\psi^{\prime }}_{\text{ref}}|{\psi^{\prime }}_{\text{ref}}\rangle +\langle {\psi^{\prime }}_{f}|{\psi^{\prime }}_{f}\rangle \\ \;+2\left|\langle {\psi^{\prime }}_{\text{ref}}|{\psi^{\prime }}_{f}\rangle \right|\cos\left(\eta -\arg\langle {\psi^{\prime }}_{\text{ref}}|{\psi^{\prime }}_{f}\rangle \right). \end{array}$$ (5)

We notice a phase shift of the interference pattern by arg $\langle {\psi^{\prime }}_{\text{ref}}|{\psi^{\prime }}_{f}\rangle$. This phase shifts corresponds to the Pancharatnam connection [5] between the state $|{\psi^{\prime }}_{f}\rangle$ and the state $|{\psi^{\prime }}_{\text{ref}}\rangle =|q\rangle \;\langle q|\psi_{t}\rangle =|q\rangle$ from which we can extract the geometric phase. Explicitly we obtain

$$\begin{array}{c} \phi =\arg\langle {\psi^{\prime }}_{\text{ref}}|{\psi^{\prime }}_{f}\rangle \\ \;=\frac{\phi_{1}+\phi_{2}}{2}-\arctan\left[\tan\left(\frac{\Delta \phi }{2}\right)\left(\frac{1-\sqrt{T}}{1+\sqrt{T}}\right)\right], \end{array}$$ (6)

where Δ*ϕ* ≡ *ϕ*_2_−*ϕ*_1_. The geometric phase is defined as [6]

$$\phi_{g}\equiv \arg\langle {\psi^{\prime }}_{\text{ref}}|{\psi^{\prime }}_{f}\rangle -\phi_{d},$$ (7)

where *ϕ*_d_ denotes the dynamical part. From Refs. [13] and [15] we know that the dynamical part stemming from the phase shifter PS2 is given by a weighted sum of the phase shifts *ϕ*_1_ and *ϕ*_2_ with the weights depending on the transmission coeficient *T*. In particular we have

$$\phi_{d}=\frac{\phi_{1}+T\phi_{2}}{1+T},$$ (8)

which vanishes by an appropriate choice of phase shifts and transmission, i. e., *ϕ*_d_ = 0 for *ϕ*_1_/*ϕ*_2_ = −*T*.

**Fig. 2:**
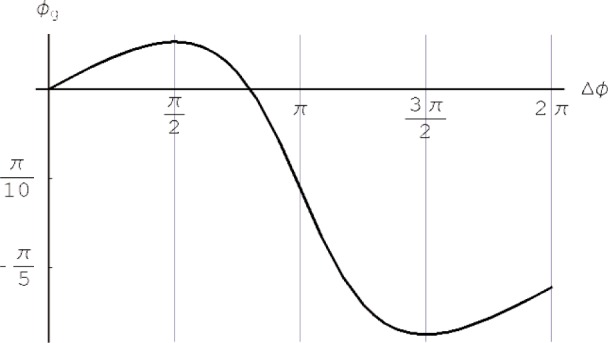
Geometric phase *ϕ*_g_ [rad] in dependence on the relative phase shift Δ*ϕ* [rad] for *T* = 1/8.

By varying the relative phase Δ*ϕ* from 0 to 2π and setting *ϕ*_d_ = 0 the geometric phase *ϕ*_g_ can be plotted over Δ*ϕ* (Fig. 2).

## 4. Bloch-Sphere Description

For every two-level system we can use the *Bloch-sphere* for depicting the state vectors and evolutions thereof as points and curves on a sphere. Then the results obtained above, i. e., the shift of the interference pattern in Eq. (5) without dynamical contributions, should be equal to the (oriented) surface area enclosed by the paths of the state vectors on the Bloch-sphere, or, equivalently, to the solid angle traced out by the state vectors as seem from the origin of the sphere.

**Fig. 3:**
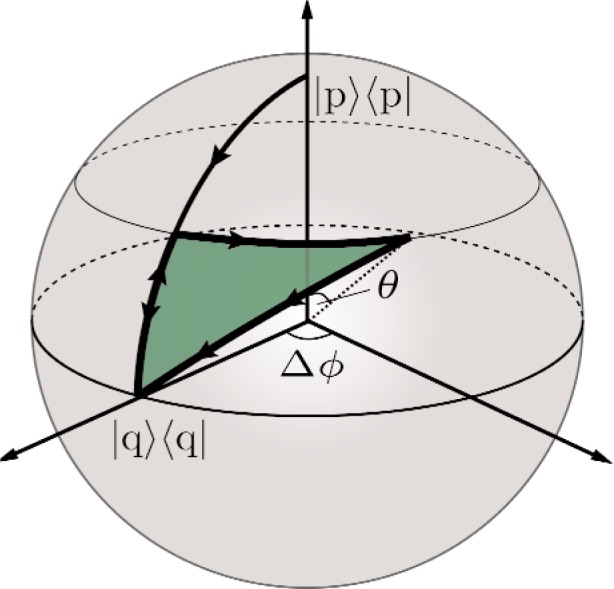
Path of the state in an interferometer on the Bloch sphere representing the 2-level system (upper path |*p*〉 〈*p*| and lower path |*p*′〉 〈*p*′|).

As we can observe in Fig. 3, the north pole of the sphere can be identified with a state with well known path, i. e., an eigenstate of the observable |*p*〉 〈*p*|. After the beam splitter BS1 the state |*ψ*_t_〉 evolves to an equal superposition of upper path and lower path, therefore the evolution on the Bloch sphere is given by a geodesic from the north pole to the equatorial line (the particular point on the equator is arbitrary due to the arbitrary choice of the phases of the basis vectors).

The absorber changes the weights of the superposed basis states, in particular for the extremal values of *T* parameterized by the angle *θ* with *T* = tan^2^ *θ*/2, we end up either again with an equally weighted superposition for no absorption (*T* = 1 or *θ* = π/2) or the state is now on the north pole for total absorption (*T* = 0 or *θ* = 0), since in the latter scenario we know the particle has taken the upper path when detecting a neutron in *D*_0_. For *T* ∈ (0, 1) the state is encoded as a point on the geodesic from the north pole to the equatorial line.

Due to the phase shifter PS2 we obtain a relative phase shift between the superposing states of Δ*ϕ* = *ϕ*_2_−*ϕ*_1_:

$$\frac{1}{\sqrt{2}}\left(|p\rangle +|p^{⊥}\rangle \right)\mapsto \frac{1}{\sqrt{2}}\left(|p\rangle +e^{i\Delta \phi }|p^{⊥}\rangle \right).$$ (9)

This can be depicted as an evolution along a circle of latitude on the Bloch sphere with periodicity of 2π.

The recombination at BS2 followed by the detection of the forward beam in *D*_0_ is represented as a projection to the starting point on the equatorial line, i. e., we have to close the curve associated with the evolution of the state by a geodesic to the point |*q*〉 〈*q*| on the sphere as discussed for non-cyclic paths in Sec. 2. As for the reference state |*ψ*_ref_〉 we note that the phase shift of *η* has no impact on the position of the state on the Bloch sphere, it stays at the north pole. Due to the recombination at BS2 and the detection the state is also projected to |*q*〉 〈*q*| contributing to the forward beam incident to the detector *D*_0_.

**Fig. 4:**
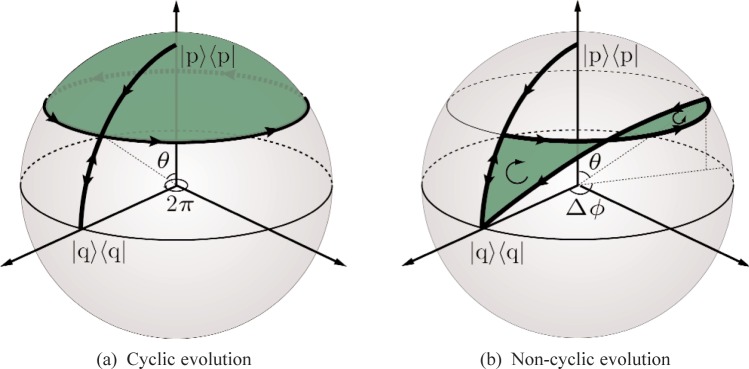
Paths on the Bloch sphere corresponding to the evolution of the state in the splitbeam experiment.

The paths are depicted in Fig. 4 in detail for cyclic Fig. 4(a) as well as non-cyclic evolution, Fig. 4(b). For a relative phase difference greater than π/2 we have to take the direction of the loops into account. In Fig. 4(b) the first loop is traversed clockwise, whereas the second loop is traversed counterclockwise yielding a positive or negative contribution to the geometric phase, respectively.

The numerical calculation of the surface area *F* enclosed by the path traversed by the neutron is straightforward by evaluating the solid angle Ω = ∫*_F_* sin *θ* d(Δ*ϕ*)d*θ* via a surface integral and using *ϕ*_g_ = −Ω/2. For the cyclic case this integral can be solved easily by calculating the segment on the sphere according to Fig. 4(a) to obtain *ϕ*_g_ = −Ω/2 = π(cos*θ* −1). For the non-cyclic case no analytic expression has been found to compare the results with the phase shift of the interference fringes appearing in Eq. (5). However, the numerical results are equivalent and agree with Fig. 2.

## 5. Experimental Results

**Fig. 5:**
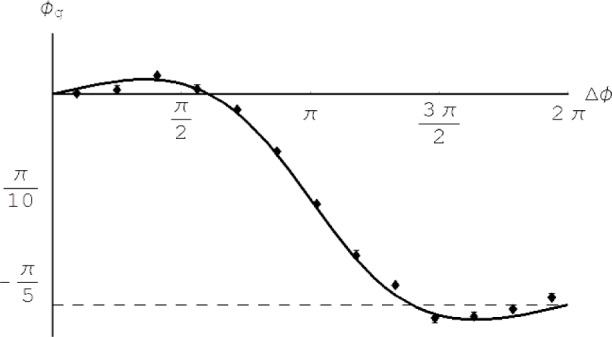
Experimental verification of the spatial geometric phase using a neutron interferometry setup.

For an experimental test of the spatial geometric beam we have used the double-loop perfect-crystal-interferometer installed at the S18-beamline at the high-flux reactor at the Institut Laue-Langevin, Grenoble [17]. A schematic view of the setup is shown in Fig. 1. Before falling onto the skew-symmetric interferometer the incident neutron beam was collimated and monochromatized by the 220-Bragg reflection of a Si perfect crystal monochromator placed in the thermal neutron guide H25. The wavelength was tuned to give a mean value of *λ*_0_ = 0.2715 nm. To eliminate the higher harmonics we have used prism-shaped silicon wedges. The beam cross-section was confined to (5 × 5) mm^2^ and an isothermal box enclosed the interferometer to achieve reasonable thermal environmental isolation. For the phase shifters parallel sided aluminium plates have been used: 4 mm inserted in the first loop as PS1 and 4 mm and 0.5 mm, respectively, in the second loop for PS2 yielding a ratio of 1/8 for *ϕ*_1_/*ϕ*_2_. To avoid dynamical phase contributions a gadolinium solution with *T* = 0.118 [19] has been used as an absorber. For a comparison with the theoretically predicted values one has to keep in mind that the contrast reflecting the coherence properties is different between each of the beams in the interferometer. Accounting for this experimental fact in the theoretical derivation of the geometric phase we notice a slightly flattened curve in Fig. 5 compared to Fig. 2. Nevertheless, one can recognize the increase in geometric phase for Δ*ϕ* ∈ [0, π/2] due to the positively oriented surface followed by a decrease due to appearance of a counter-clockwise traversed loop on the sphere yielding a negative phase contribution.

## 6. Conclusions

In summary we have shown that one can ascribe a geometric phase not only to spin evolutions of neutrons, but also to evolutions in the spatial degrees of freedom of neutrons in an interferometric setup. This equivalence is evident from the description of both cases via state vectors in a two dimensional Hilbert space. However, there have been arguments contra to the experimental verification in [13] which we believe can be settled in favour of a geometric phase appearing in the setup described above. The twofold calculations of the geometric either in terms of a shift in the interference fringes or via surface integrals in an abstract state space allows for a geometric interpretation of the obtained phase shift.
